# Mental Diseases

**Published:** 1914-06

**Authors:** 


**Mental Diseases.** By R. H. Cole, M.D. Pp. x, 343. London: University of London Press. 1913. Price 10s. 6d. net.

If there be one branch of medicine which does not lend itself, after the mode of the late Pelissier, to "potting," it is that of mental diseases. Should, as in the present case, the object be to give the medical student and general practitioner such a sufficient knowledge of the subject as the many other claims on their time for study allows, the plan preferably to be pursued is that of affording a general and equable survey, neither curt nor discursive, avoiding minutiæ where possible on the one hand, and superficial or scant treatment of any parts on the other. The most ineffective purpose for this class of readers is that of attempting in a short work to embrace all obtainable details and to indicate every current speculation. This can only be done by sacrificing literary style, and projecting bald statements at the unfortunate reader as with a pea-shooter. Many works on insanity have faults of one kind or another, not seldom a want of evidence as to any reason for having been written at all. We do not, however, recall any book previous to this whose author has so failed to realise that such a difficult subject is only to be presented by one who, having absorbed the contents of his pigeon-holes, is able to transmute them into a product the details of which are harmoniously connected, imaginatively treated, graphically illustrated, and decked with some show of literary polish, The reader needs to be guided from one statement to another, his attention to be courted, his reflection stimulated, his patience neither exhausted by excessive minutiæ nor tempted to flag by obvious superficiality. This book conveys to us nothing of an author's art. It reads merely as a slight amplification of the contents of a note-book fully stocked with abstracts of current literature. Of the author's views or investigations we find nothing, there is no personal note from beginning to end. As a result of this method of presentation we opine that, although in the way of information to the reader Dr. Cole has been punctilious in leaving nothing out, we are doubtful whether he has been so successful in putting anything in.

To instance the author's "conciseness" let us take the paragraph on obsessive ideas. We find these described as "generally due to emotional stress, with defective volition." The subject is dismissed in less than five lines. At page 44, on the origin of delusions, we read: "The retardation of ideas and painful emotion in depression produce a splitting-off of certain ideational complexes from the general personality." Original or quoted, this is pretty strong meat for psychological babes! As a specimen of the spasmodic, jerky style of writing throughout the book, take the section on the Etiology of G.P. Here are twenty-five statements, separated by full stops, in sixty-nine lines. A little later another paragraph contains fourteen statements in thirty-six lines. The effect of this piecemeal method of writing is fatiguing to the last degree.

The book seems to us to present possibilities of use to the Asylum Medical Officer as an index or work of brief reference. Were its other qualities at all proportionate to the careful completeness of its compilation it would be a good book.

